# Bioinformatics evaluation of anticancer properties of GP63 protein-derived peptides on MMP2 protein of melanoma cancer

**DOI:** 10.1016/j.jpi.2023.100190

**Published:** 2023-01-12

**Authors:** Fatemeh Sharifi, Iraj Sharifi, Zahra Babaei, Sodabeh Alahdin, Ali Afgar

**Affiliations:** a Research Center of Tropical and Infectious Diseases, Kerman University of Medical Sciences, Kerman, Iran; b Leishmaniasis Research Center, Kerman University of Medical Sciences, Kerman, Iran; c Student Research Committee, Kerman University of Medical Sciences, Kerman, Iran; d Research Center for Hydatid Disease in Iran, Kerman University of Medical Sciences, Kerman, Iran

**Keywords:** Leishmanolysin, Matrix metalloproteases, *Leishmania*, *In silico*, Anticancer, Peptide, Protein–Ligand Interaction Profiler, CL, cutaneous leishmaniasis, VL, visceral leishmaniasis, GP63, Glycoprotein 63, ROS, reactive oxygen species formation, MSP, major surface protease, MMPs, matrix metalloproteases, ACPs, anticancer peptides, SVM, Support Vector Machine, kNN, k-Nearest Neighbors, MD, molecular dynamics, PDB, Protein Data Bank, CASTp, Computed Atlas of Surface Topography of proteins, PLIP, Protein–Ligand Interaction Profiler

## Abstract

**Background:**

GP63, also known as Leishmanolysin, is a multifunctional virulence factor abundant on the surface of *Leishmania* spp. small peptides with anticancer capabilities that are selective and toxic to cancer cells are known as anticancer peptides. We aimed to demonstrate the activity of GP63 and its anticancer properties on melanoma using a range of *in silico* tools and screening methods to identify predicted and designed anticancer peptides.

**Methods:**

Various *in silico* modeling methodologies are used to establish the three-dimensional (3D) structure of GP63. Refinement and re-evaluation of the modeled structures and the built models' quality evaluated using the different docking used to find the interacting amino acids between MMP2 and GP63 and its anticancer peptides. AntiCP2.0 is used for screening anticancer peptides. 2D interaction plots of protein–ligand complexes evaluated by Protein–Ligand Interaction Profiler server. It is for the first time that used anticancer peptides of GP63 and the predicted and designed peptides.

**Results:**

We used 3 peptides of GP63 based on the AntiCP 2.0 server with scores of 0.63, 0.53, and 0.49, and common peptides of GP63/MMP2 (continues peptide: mean the completely selected peptide after docking with non-anticancer effect, predicted with 0.58 score and designed peptides with 0.47 and 0.45 scores by AntiCP 2.0 server).

**Conclusions:**

The antileishmanial and anticancer peptide research topics exemplify the multidisciplinary nature of peptide research. The advancement of therapeutics targeting cancer and/or *Leishmania* requires an interconnected research strategy shown in this work.

## Introduction

Leishmaniasis constitutes a cluster of diseases caused by 20 diverse parasitic protozoan species of the *Leishmania* genus. Over 1 billion individuals in 98 countries are at risk of infection, with 1.5 million new cases and 20 000–40 000 deaths reported each year.^1^ The World Health Organization estimates that 1.3 million cases of cutaneous leishmaniasis (CL) and 300 000 cases of visceral leishmaniasis (VL) have been reported in the last 5 years.^2^ The multifunctional virulence factor Glycoprotein 63 (GP63) has been characterized as being abundant on the surface of *Leishmania* spp.^3^ It is the main enzyme in parasite replication, promastigote binding to and internalization in macrophages, and attenuation of reactive oxygen species formation (ROS), which favors amastigote proliferation.^3^^,^^4^ This gene with higher copy numbers encodes a surface protein, and is an essential virulence factor in *Leishmani*a, favoring rapid migration, internalization, and survival in host macrophages.^5^^,^^6^

The GP63 protease, also known as leishmanolysin or major surface protease (MSP), was first found as the primary surface antigen of numerous *Leishmania* promastigote species in 1980.^7^ GP63 shields parasites from lysosomal cytolysis and macrophage degradative activities.^8^ It can also protect liposome-encapsulated proteins from macrophage phagolysosomal degradation.^9^ GP63 is vital for macrophage binding to macrophages' survival and replication.^7^ GP63 is one of the hopeful candidates for a subunit vaccine against leishmaniasis. Zhang *et al*. used GP63 protein as pre-selected antigens to explore effective multi-epitopes DNA prime-protein boost vaccines against visceral leishmaniasis (VL) and selected the amino acid sequences according to the *in silico* analysis.^10^

Melanoma is an especially lethal type of skin cancer, accounting for 75% of all skin cancer deaths despite causing just 4% of all skin cancers. The melanoma-specific genetic profile has yet to be discovered, as have early indications of melanoma initiation.^11^ Due to metastasis to other parts of the body, such as the brain, lymph nodes, lungs, liver, or bone, melanoma, an exceedingly aggressive disease, is responsible for most skin cancer-related deaths.^12^ Proteolysis in the pericellular and stromal compartments plays a vital role in the invasion process, and many proteases, such as matrix metalloproteases (MMPs), are known to be mediators of melanoma growth.^13^ MMPs are a more prominent family of proteases that degrade various extracellular matrix proteins and process many bioactive molecules. They are involved in cell membrane receptors' cleavage, apoptosis release, and cytokine/chemokine inactivation. MMPs also play a pivotal role in cell activities, including growth, differentiation, migration, angiogenesis, programmed cell death, and host defense mechanisms.^14^ MMPs, help cancer spread by promoting cell proliferation, migration, invasion, metastasis, and angiogenesis. Metalloproteases are enzymes required for cell proliferation, differentiation, extracellular remodeling, and cell migration in physiological and pathological processes. MMP2 and MMP9 are 2 enzymes that cleave collagen type IV, and type V, and denature collagens from the basement membrane and gelatins. MMP relative expression levels seem to increase with tumor growth, thus far and many reports have connected greater MMP2 and MMP9 levels to enhancing metastatic cells. MMP2 expression is elevated in various human cancers, including colon, pancreatic, prostate, bladder, skin, breast, and ovarian carcinomas. Active MMP2 is closely linked to tumor cell invasion and metastasis.^15^, ^16^, ^17^, ^18^ High MMP2 secretions damage the extracellular matrix and basement membrane, allowing cancer cells to migrate to distant organs.^19^ The collagenase matrix metalloproteinase 2 (MMP2) facilitates tumor growth and invasion by degrading the tumor tissue's extracellular matrix. MMP2 (gelatinase A) plays a crucial function in tumor growth because of its ability to break down collagen IV found in the basement membrane.^33^

*Leishmania* spp. are intracellular parasites with diverse pathways for surviving and multiplying within their host's phagocytic mononuclear cell structure. Since the host recognizes most *Leishmania* antigens, antibodies and/or cellular immune responses generated during host infection. However, some antigens' immune response does not provide protection.^27^ Different antigens (polyvalent) can induce a defensive immune response in most of the population, and that can be generated in large quantities are needed to diagnose our leishmaniasis vaccine.^28^

GP63 is located on the surface of the promastigote stage in the gut of the biological sand fly vector engulfed by host macrophages, transforming into the pathogenic amastigote stage (clinical stage or Leishman bodies). Gp63 is presumably playing a pivotal role in many stages of infection,^29^ i.e., their entry into macrophages^30^ and intra-lysosomal survival.^31^

The experimental identification and characterization of novel anticancer peptides are time-consuming and labor-intensive; hence, a preliminary analysis is required to decrease time, workforce, and manufacturing costs, critical in preclinical evaluations of their adverse impacts.^34^ When a compound is obtained in the pharmaceutical industry, many chemical changes are performed that must be examined using biological testing because there is a requirement to improve specific qualities. This number of molecules can be reduced if a few are previously selected using *in silico* analysis.^35^ Furthermore, these analyses complement the natural results and are currently used to identify pharmacological targets, lead molecules, active compounds, and preclinical tests.^36^ Thus, *in silico* process will play a critical role in discovering and developing new molecules that become a competitive advantage among pharmaceutical companies and will differentiate those who apply them and those who do not.^37^ Since identifying and screening possible anticancer peptides in the lab is time-consuming, expensive, and labor-intensive, advanced *in silico* tools are required. Many designing tools that can forecast and design novel ACPs have been developed.^36^ Inferring the natural function of proteins from their 3D forms is one of the most attractive topics in modern medicine and biology. With so many proteins and isoforms in an organism, establishing the 3D structure of a protein through experimental methods can be difficult.^38^ Bioinformatics methods are becoming more popular due to their advantages over experimental procedures (cost-effectiveness, acceptable accuracy, time-saving, and labor).^39^ Although the top techniques for predicting the 3D structure of proteins are considered in bioinformatics modeling, it has been discovered that the projected models might differ significantly from their native structures.^40^

Small peptides with anticancer capabilities that are selective and poisonous to cancer cells are known as anticancer peptides (ACPs). Because of their significant penetration, selectivity, and simplicity of modification, synthetic peptide-based medicines and vaccines are a potential class of therapeutic agents. ACPs must identify from peptide sequences by drug developers and researchers. Over 7000 naturally occurring peptides with various bioactivities have been identified in the recent decade (antibacterial, anticancer, antifungal, antiviral, and tumor-homing).^20^ Experimental strategies for identifying and developing novel ACPs are expensive and time-consuming. Several machine learning methods have already been employed for ACP design, such as Support Vector Machine (SVM),^21^ k-Nearest Neighbors (kNN),^21^ Random Forest (RF),^21^ and generalized/probabilistic neural networks.^22^ However, many user-friendly web servers, including AntiCP 2.0, have been established to assist the scientific community in predicting and designing highly useful ACPs.^23^

The 3D structure of *Leishmania major*, Leishmanolysin protein, GP63, was developed using homologous modeling and configured using molecular dynamics (MD). The 3D structure of the GP63 protein was modeled using comparative modeling, with the X-ray crystal structure of the *Leishmania major* GP63 (PDB code: 1LML) serving as a template.^24^ The molecular docking method predicts the desired orientation of small organic molecules (ligands) within biological macromolecules (proteins). Protein configurations and ligand–protein interactions have also been successfully predicted using computational approaches.

To our knowledge, this is the first time that, based on the docking affinity, a leishmanial GP63 peptide and a synthesized peptide was designed which led to the discovery of ACPs. With the advancement of bioinformatics in molecular modeling, we aimed to demonstrate the activity of GP63 and its anticancer peptides on melanoma using a range of *in silico* tools and screening methods to identify predicted and designed anticancer peptides. Further, we tried to dissect its mechanism of action by *in silico* approaches with the *Leishmania* promastigote-specific protein, GP63, on host MMP2.

## Materials and methods

### Sequence similarity/identity evaluation

The amino acid sequence of *Leishmania major* GP63 was retrieved from the UniProt (Universal Protein Resource) knowledge base at http://www.uniprot.org/ with the accession number A0A0S2UX54 and stored in FASTA format for further analysis. After determining the degree of homology, differences among amino acid positions, and the frequency-based difference, a COBALT blast (multiple alignments) was performed with the blosum 80. The GP63 sequence was used to query the Protein Data Bank (PDB) proteins database using BLAST at http://blast.ncbi.nlm.nih.gov/Blast.cgi to discover an appropriate template structure for homology modeling predictions.

### Topology and prediction of secondary structures of proteins

Nowadays, effective methods are called the self-optimized prediction method designed to improve the success rate of predicting the secondary structure of proteins. SOPMA (https://npsa-prabi.ibcp.fr/cgi-bin/npsa_automat.pl?page=/NPSA/npsa_sopma.html) and PSIPRED (http://bioinf.cs.ucl.ac.uk/psipred/&uuid=f2b9e69a-5fde-11ec-97a3-00163e100d53) accurately predicted the number of random coils, ß-sheet/extended strand, and α-helix conformations in the secondary structure.

### Multi-template homology modeling

Based on the 3D structure prediction algorithms of each software or web server, protein structure based on fold/or multiple threading approaches was utilized to predict the parasite's 3D structure. At first, all GP63 sequences were downloaded from multiply aligned sequences by Mega 7 software and modeled using SWISS-MODEL online software at https://swissmodel.expasy.org.

I-TASSER (Iterative Threading Assembly Refinement; http://zhanglab.ccmb.med.umich.edu/ITASSER/), the most accurate predictor for protein structure/function, which used advanced algorithms was also applied. The server finds the structural patterns from the Protein Data Bank (PDB) using the multiple threading LOMETS.

Furthermore, ModWeb was employed to predict the 3D structure of the target proteins and loops correction at https://modbase.compbio.ucsf.edu/modweb/. This server predicts the most similar 3D structure of the target proteins based on the SwissProt database experimental data. Graphically, all models were structurally aligned by chimera-1.14 software, and meticulous evaluation was performed to determine the conservation points and positions of the amino acids in the 3D structures.

### Refinement and re-evaluation of the modeled structures

The refinement process of the 3D models was used to assess global quality, compare refined and original models, optimize the hydrogen-bonding network, and predict the local quality (per-residue error) by the i3Drefine server at http://sysbio.rnet.missouri.edu/3Drefine/. First, the obtained structures were inserted into the ModLoop server (https://modbase.compbio.ucsf.edu/modloop/), and loop regions spanning the amino acids were remodeled. 3D refine is an entirely free program under Linux that does not require registration. Next, using the Rampage server (https://zlab.umassmed.edu/bu/rama/), the predicted structures and, in particular, the Ramachandran plot were analyzed.

### Evaluation of the quality of the models

For assessing the predicted GP63 structures' quality, the above online servers' obtained PDB files uploaded separately on the QMEAN (Qualitative Model Energy Analysis; http://swissmodel.expasy.org/qmean/cgi/index.cgi) and PROSA servers (https://prosa.services.came.sbg.ac.at/prosa.phpto). These servers estimate the predicted models' quality and recognize the possible unreliable regions.

### Prediction of protein functional residues

HotSpot Wizard 3^25^ at https://loschmidt.chemi.muni.cz/hotspotwizard/ was working to predict the available hot spots/residues of the metalloprotease protein structure. This program provides predictions by combining structural, functional, and evolutionary data from various bioinformatics sources and computational methods. However, by anticipating fluctuating residues in pockets and access tunnels, this web server can determine available hotSpots.

### Detecting structural pockets and cavities

Protein structures' geometric and topological characteristics, such as interior cavities, transverse channels, and surface pockets, are critical for understanding their functions. CASTp (Computed Atlas of Surface Topography of proteins), a web server at http://sts.bioe.uic.edu/castp/calculation.html, measures holes in the protein's 3D structure, including surface pockets as well as holes buried inside the protein.

### Docking and virtual screening to find the interacting amino acids between MMP2 and GP63

For exploring the interaction between GP63 and metalloprotease receptors, molecular docking was accomplished using 2 servers, ClusPro server (https://cluspro.org/login.php?redir=/queue.php) and HDOCK servers. It is worth noting that the optimal docking model was chosen based on its low energy. ClusPro is a current docking method known as a fast algorithm for filtering docking confirmation by simple scoring function and yielding structures with good surface complementary. The electrostatic energies are supplied in the table with the downloaded models, and the free energy selects complexes with the least desolation. These equations generated by the server were used to calculate cluster scores.^20^ By applying the balanced approach, the energy was computed as coefficient wattage using the formula E = 0.40Erep + --0.40Eatt + 600Eelec + 1.00EDARS. The HDOCK server used peptide-protein docking to measure the interaction strength between the MMP2 binding site and GP63 peptides. The difference in location of the docked peptides was also determined using an RMSD technique. For all docking runs, the default parameters are used. The HDOCK server employs an ITScore-PP iterative knowledge-based scoring system (Docking score).^26^^,^^27^

Finally, a docking grid is employed to calculate an optimal box size and maximize the interaction accuracy among ligands and GP63 protein. This prediction may occur based on the selected residues introduced by CASTp (http://sts.bioe.uic.edu/castp/index.html?2was) and POCASA (http://g6altair.sci.hokudai.ac.jp/g6/service/pocasa/) servers.

### Screening the anticancer peptides

The peptides were then closely examined for the presence of a particular amount of anticancer-promoting amino acids. The anticancer properties of the selected antileishmanial peptides were then assessed using the AntiCP website. The 5-fold cross-validation method was used to train and validate the models, and the validation dataset was used to evaluate their performance.

The best models introduced in the AntiCP 2.0 webserver, were found at https://webs.iiitd.edu.in/raghava/anticp2. The standalone version of the program is available as a GitHub kit and a Docker container. Users may upload a peptide, and the server will produce all of the peptide's single substitution mutants. Aside from creating mutations, the server will also indicate whether the prediction is AntiCP or non-AntiCP. The server measures important physicochemical properties in a tabular format.

Furthermore, scanning several peptides at once allows the user to discover novel ACPs. A virtual screening tool has been implemented for this purpose, which requires the user to upload several peptide sequences in FASTA format. Another helpful technique is a protein scan, which can identify putative ACP regions in proteins. In PREDICT module, the anticancer potency of the submitted peptides was predicted. Users can submit multiple peptides in FASTA format in the box or upload the same file. The server will provide the result in "ACP" or "non-ACP," along with the prediction score and physiochemical properties selected during submission. Finally, AntiCP's 'Design-Peptide' module indicates single amino-terminal peptide mutations that may improve this peptide's anticancer function.

### Docking and virtual screening between MMP2 and predicted peptides from GP63 interaction and AntiCP web server

Initially, GP63 was placed in several sequences at the anticancer site (AntiCP 2.0 webserver), and the peptides with anticancer properties were scored and modeled separately with I-TASSER, and then docked with MMP2. After docking GP63 with MMP2, common peptides between the 2 were selected and modeled as a continuous peptide with I-TASSER and then docked with MMP2. These common peptides are then introduced into the anticancer site to investigate the anticancer properties (according to the protocol). Next, the peptide, which had anticancer properties, was selected and modeled on the I-TASSER site and then docked. In the next step, the common peptide was designed continuously (according to the protocol) at the AntiCP 2.0 webserver for anticancer properties. After designing the anticancer by the webserver, peptides with anticancer properties were selected and modeled on the I-TASSER webserver and then docked.

### 2D interaction plots of protein–ligand complexes

After the docking procedure, the ligand interaction diagrams are evaluated by Protein–Ligand Interaction Profiler (PLIP) server (https://plip-tool.biotec.tu-dresden.de/plip-web/plip/index). Indeed, specific amino acids involved in the protein–protein interaction, MMP2 ligands interaction plots, and potential inhibitors were identified. 2D interaction diagrams describe highly hydrophobic cavities consisting of several adjacent hydrophobic residues and ligand-cavity hydrogen bonds. Hydrophobic interactions hydrogen bonds are the fundamental contributing forces to the binding energy of the ligand and target protein.

## Results

### Sequence similarity/identity evaluation and blast search

The protein sequence of GP63 was obtained from the UniProt knowledge base with the I.D. code of UniProtKB - P08148 (GP63_LEIMA) for *in silico* analyses. Comprising 602 amino acids, the BLAST search against non-redundant protein sequences database (100.00%, XP_001681379.1 and 69.39, GET86505.1) and PDB database (97.49% with 1LML) Using this sequence as a query, identity sequences produced 89.29%–100% query coverage and 100% per identification with GP63, leishmanolysin [*Leishmania major* strain Friedlin; NCBI: txid347515] and GP63 [*Leishmania turanica*].

### Topology and prediction of secondary structures of proteins

**Fig. 1:**
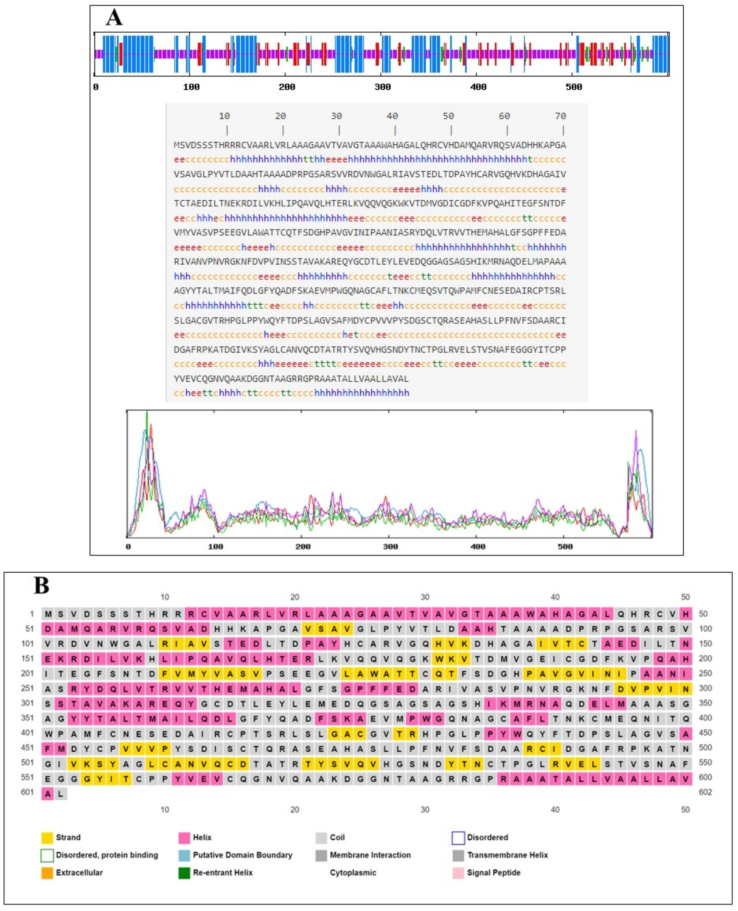
(A) SOPMA secondary structure prediction of GP63 (Parameters: window width: 17; similarity threshold: 8; the number of states: 4). (B) Secondary structure determination of GP63 by PSIPRED server showing the amino acid residues involved in forming -helix (cylinders), -sheet (arrow), and random coil (continuous line).

Protein secondary structure predicted by the SOPMA server indicates that alpha helixes consist of the most significant portion (30.07%), random coils place second (51.33%), extended strands (13.95%) place third, and beta-turn (4.65%) place fourth (Fig. 1A, Table 1). Secondary structure determination of GP63 by the PSIPRED server showed alpha helixes (28.44%), strands (16.78%), and random coils (54.77%) (Fig. 1B).

**Table 1:** Calculated secondary structures by SOPMA.

GP63	
Number of amino acids	602
α-helix	30.07%
β-turn	4.65%
Extended strand	13.95%
Random coil%	51.33%

In addition, the crystallographic structure of MMP2 protein was present in NCBI accession number (NP_004521.1), and its topology and secondary structure were not performed.

### Multi-template homology modeling

**Fig. 2:**
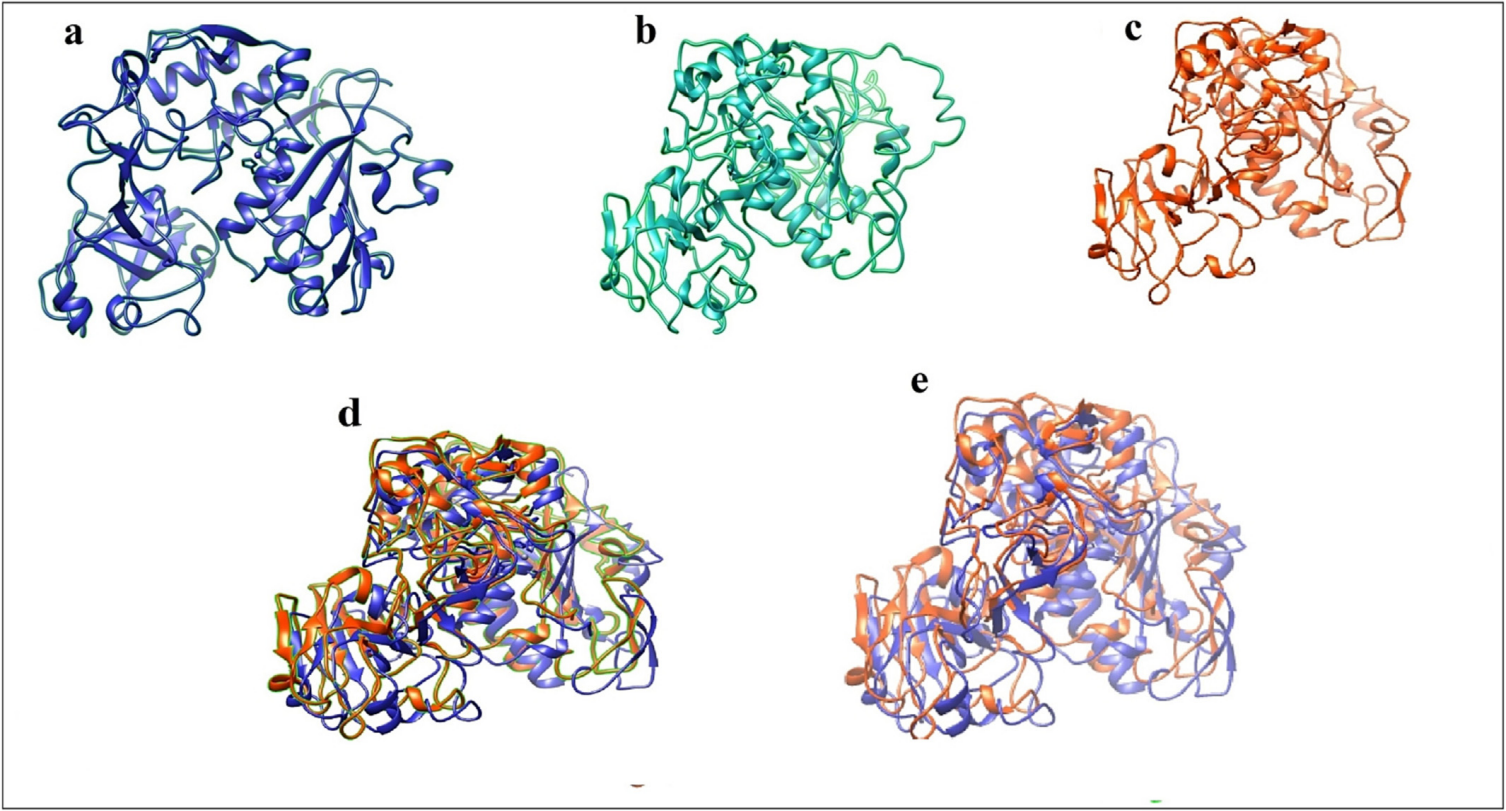
Final evaluation of the 3D structures of the refined GP63 models using Chimera software. Predicted structures of GP63 by SWISS-MODEL (a), I-TASSER MODEL (b), MODWEB MODEL (c), high homology/similarity level of SWISS-MODEL and MODWEB MODEL (d) Superposition of these structures by software (e), using PDB template Chimera was used to visualize the models.

**Fig. 3:**
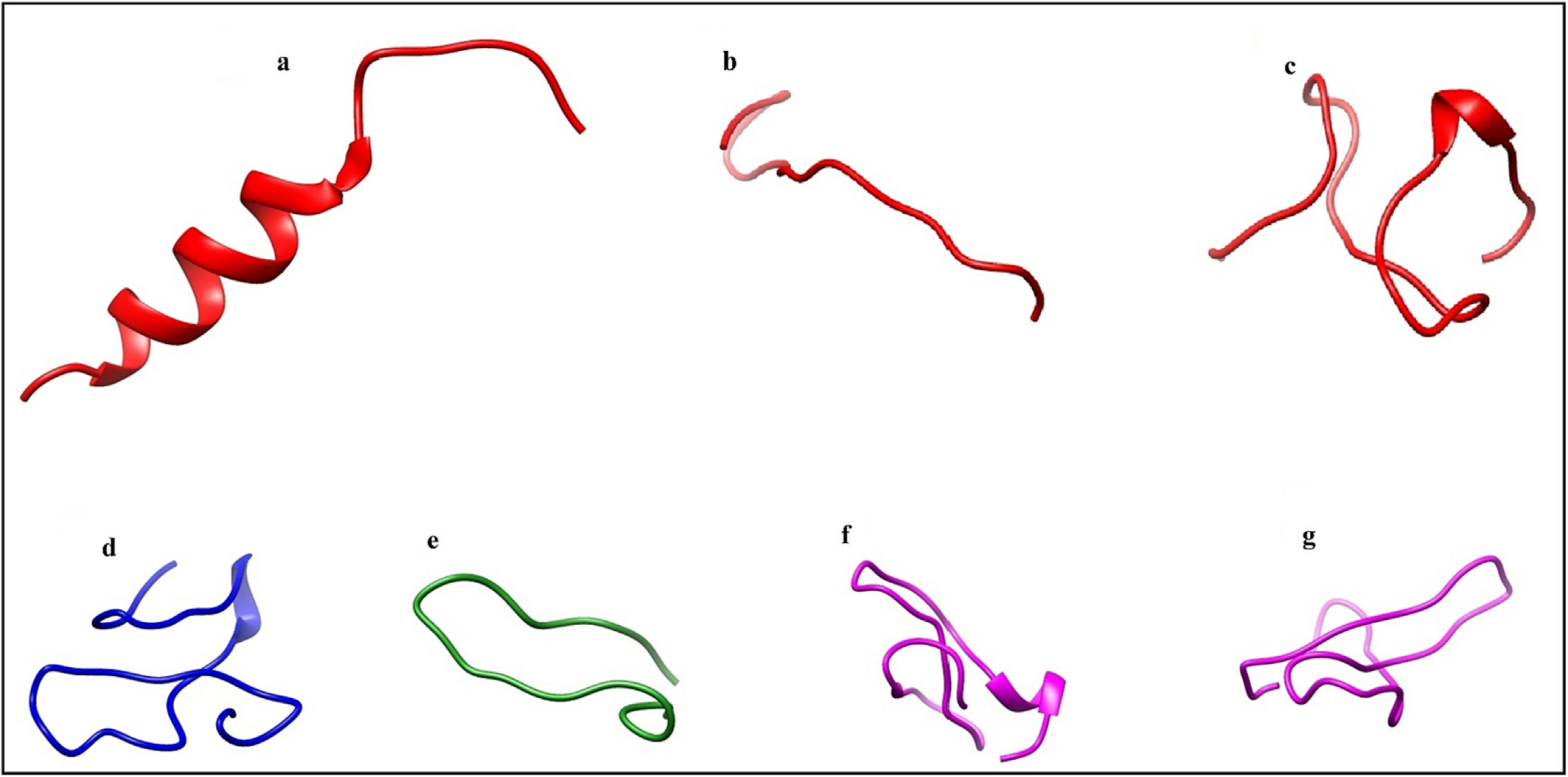
3D structures of experimentally anticancer peptides of GP63 (a, b, and c) and common peptides of GP63/MMP2, d: continuous peptide, e: predict peptide, f and h: design peptides of GP63/MMP2 by I-TASSER MODEL using Chimera software.

Experimental 3D structures were not available in common databases for GP63. Therefore, the corresponding structure for each protein is modeled using bioinformatics approaches such as homology modeling and threading by the mentioned software algorithm. Regarding the alignment results, the sequences of GP63 of *Leishmania major* were successfully modeled by several servers. Each server predicted the top structural models regarding the scoring algorithms, 5 for I-TASSER, 1–3 for ModWeb, and 2 for SWISS-MODEL (Fig. 2). As illustrated, SWISS-MODEL (a) and ModWeb (c) structures had similar 3D structure properties (d). To assess the 3D models and select the best structures for the next step, the QMEAN and PROSA servers were used to determine the Z-scores. Due to unsuitable Z-score and QMEAN, MODWEB-MODEL were excluded from the study. However, the introduced Z-scores by other servers were close (Table 2). Also, the prediction of 3D structures for various anticancer peptides of GP63 and common peptides of GP63/MMP2 designs by the I-TASSER MODEL are shown in Table 7, Fig. 3.

**Table 2:** Quality assessment scores for initial and refined GP63 models.

Severs	QMEAN	PROSA	i3Drefine	ModLoop
SWISS-MODEL	–0.66	–9.51	0.34	0.28
ITASSER-MODEL	–8.93	–6.78	–4.06	–4.15
MODWEB-MODEL	–1.83	–9.57	–0.09	0.03

### Refinement and re-evaluation of the modeled structures

**Fig. 4:**
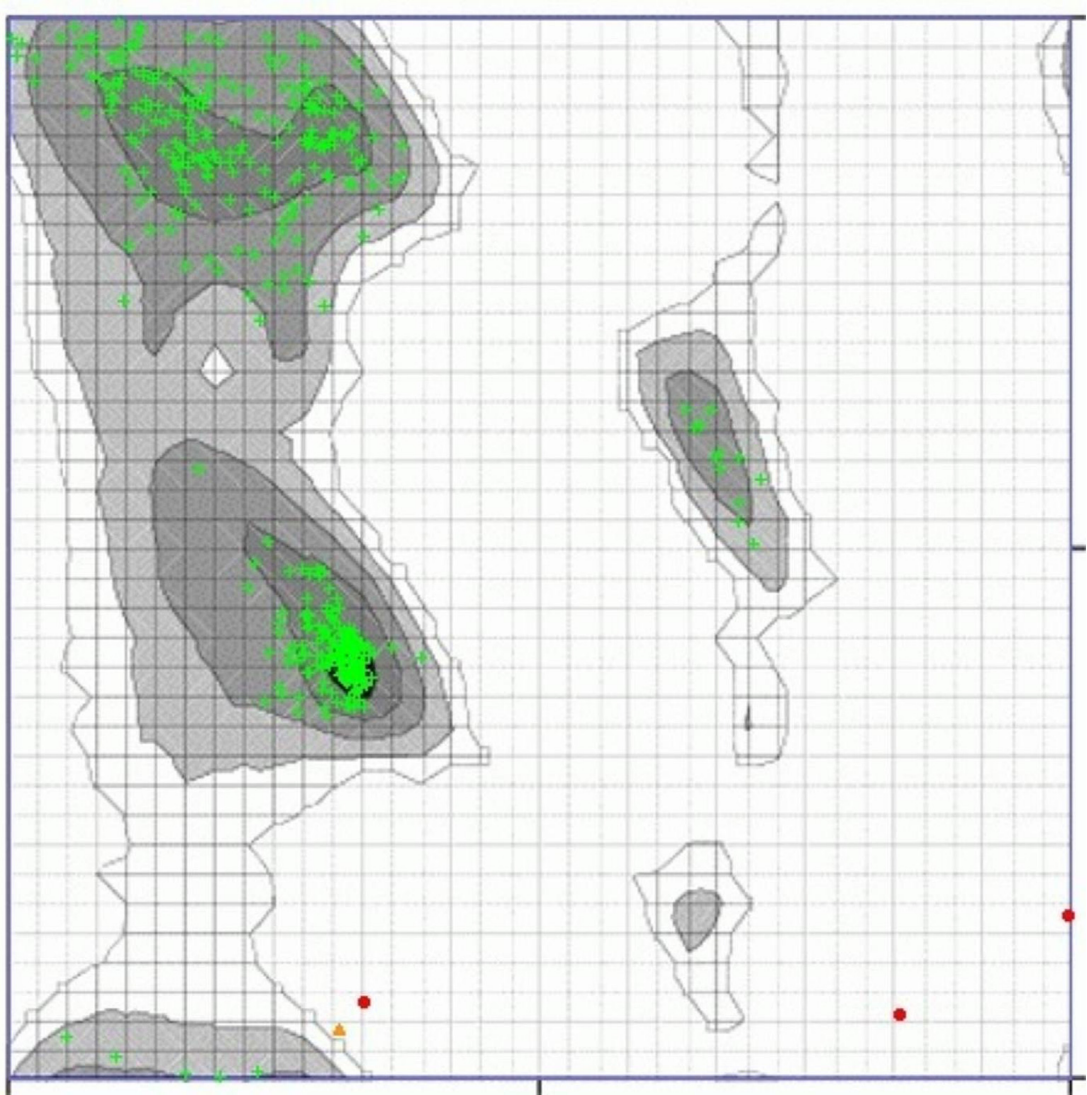
Ramachandran plot. Black, Dark Gray, Gray, and Light Gray represent Highly Preferred Conformations. Delta >= –2, White with Black Grid represents preferred conformations. –2 > Delta >= –4, White with Gray Grid represents questionable conformations. Delta < –4, Highly Preferred observations shown as GREEN Crosses: 411 (99.036%), Preferred observations shown as BROWN Triangles: 1 (0.241%), Questionable observations shown as RED Circles: 3 (0.723%).

To demonstrate predicted models of the 3D structure of proteins with exact experimental structures, whole atomic refinement, and loop modeling is required (Table 3). In the following, almost all loop regions were refined and re-evaluated by the ModLoop software. None of the modified loop files showed residue changes in the GP63 protein loop regions compared to the initial PDB files. The results of Ramachandran plot analysis for GP63 (MODWEB MODE) illustrated that the refined structures are very reliable compared to the initially predicted structures (99.036% and none of the residues are in the favored, allowed, and outlier regions, respectively) (Fig. 4) and the refining process did not change the percentage of residues in the different plot areas. There was no gap in the input sequence files as confirmed by https://bioserv.rpbs.univ-paris-diderot.fr/services/DaReUS-Loop/.

**Table 3:** Refinement and Z-score assessments of SWISS-MODEL, MODWEB, and I-TASSER MODEL by the i3Drefine server.

Server	3Drefine Score	GDT-TS	GDT-HA	RMSD (Å)	MolProbity	RWPlus
SWISS-MODEL	19 440.1	1.0000	0.9963	0.232	1.255	–100 836.841590
ITASSER-MODEL	34 602.1	1.0000	0.9975	0.152	3.484	–113 968.133393
MODWEB-MODEL	20 631.9	1.0000	0.9953	0.242	2.110	–100 001.902413

Rows are ordered by best RWPlus Score if selected. If not, rows are ordered by MolProbity Score, if selected, or by 3Drefine Score

### Prediction of protein functional residues

**Fig. 5:**
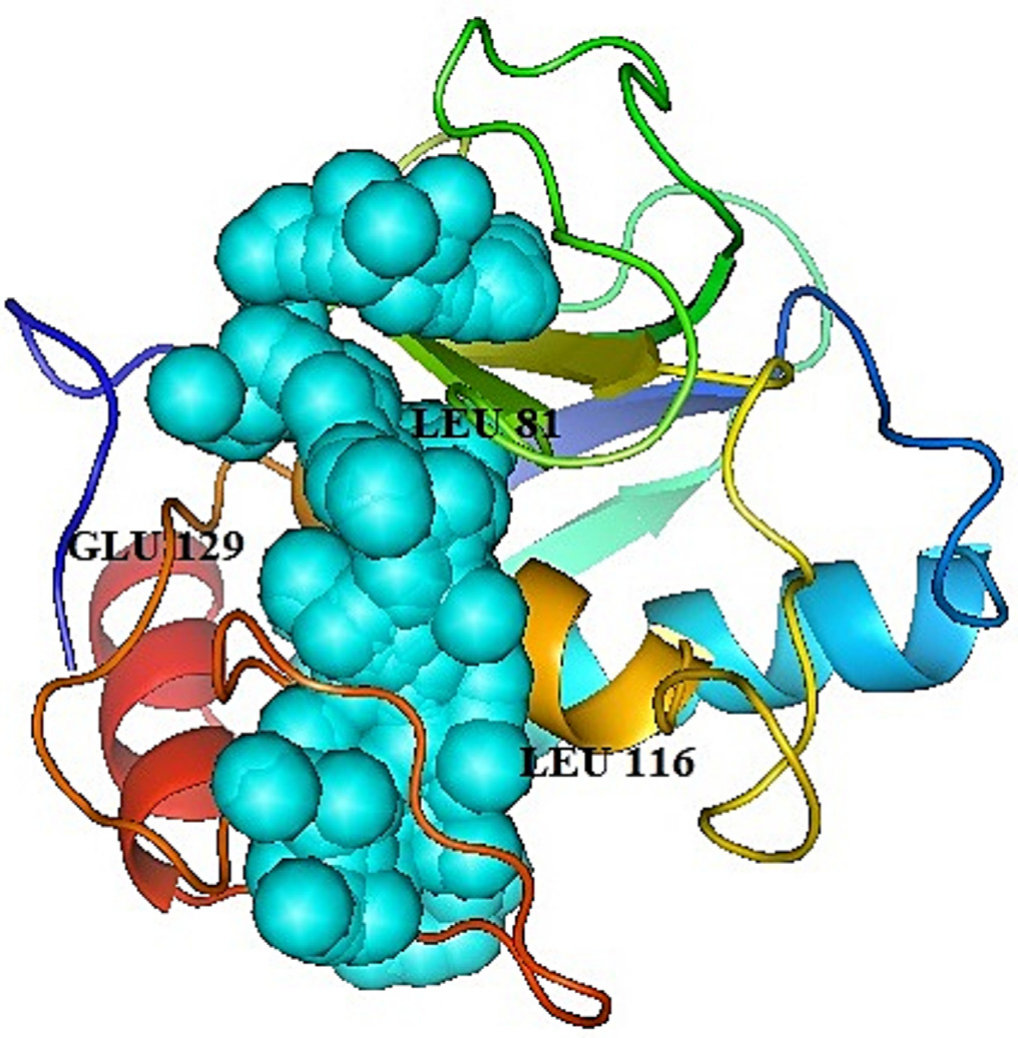
Prediction of MMP2 functional residues. As evidenced, the functional residues (3 different views: A. Glu129, B. Leu81, C. Leu116, and D. all 3) were conserved amino acids.

The files of MMP2 (3AYU) were imported into the HotSpot Wizard 3 server. The amino acids Leu 81, Leu 116, and Glu 129 were predicted as highly reliable and mutable residues situated at the accessible tunnels and/or catalytic pockets (Fig. 5).

### Detecting structural pockets and cavities

Using the Roll algorithm and detecting structural pockets and cavities, POCASA (Pocket-Cavity Search Application) program 1.1 (http://g6altair.sci.hokudai.ac.jp/g6/service/pocasa/) can anticipate binding sites. Topological and geometric structure features such as surface pockets have a critical functional impact on proteins. The CASTp-predicted volumes (http://sts.bioe.uic.edu/castp/calculation.html) for the pockets, solvent accessible surface area or volume (S.A.), and mouth M.S. area were 105.795 and 57.181. The participating amino acids of MMP2 pockets were as follows; 116, 134, 135, 136, 137, 139, 141, 142, 143, 145, 148, 149, and 151. S.A. values by POCASA 1.1 server were between Pocket 97's volume is 216, V.D. value is 656, the average V.D. is 3.04012, and Pocket 81's volume is 23, V.D. value is 53, the average V.D. is 2.33333.

### Screening for anticancer peptides

This study used 3 peptides of GP63 based on the AntiCP 2.0 server (Table 4) and common peptides of GP63/MMP2 (continues peptide: mean the whole selected peptide after docking) predicted and designed peptides by AntiCP 2.0 server. Prediction results for experimentally verified ACPs (anticancer peptides) of cancer cells in GP63, and common peptides of GP63/MMP2 are shown in Table 5.

**Table 4:** Prediction results for experimentally verified ACPs of cancer cells in GP63.

ID	Sequences	Score	Prediction	Hydrophobicity	Hydropathicity	Hydrophilicity	Charge
b	LIPQAVQLHTERLKVQQVQGKWKV	0.49	AntiCP	–0.19	–0.40	–0.08	3.50
c	EEGVLAWATTCQTFSDGHPAV	0.63	AntiCP	–0.01	–0.02	–0.26	–2.50
d	HPGLPPYWQYFTDPSLAGVSAFMDYCPVVV	0.53	AntiCP	0.08	0.28	-0.74	–1.50

**Table 5:** Prediction results for common peptides of GP63/MMP2, e: continuous peptide, f: predict peptide, g and h: design peptides of GP63/MMP2.

ID	Sequences	Score	Prediction	Hydrophobicity	Hydropathicity	Hydrophilicity	Charge
e	VPSEEGVLADQGSAGGACGVT RHPGLPVVPYSDISC	0.34	Non-AntiCP	–0.02	0.12	–0.09	–2.50
f	GACGVTRHPGLPVVPYSDISC	0.58	AntiCP	–0.00	0.33	–0.34	0.50
g	VPSEERVLADQGSAGGACGVT RHPGLPVVPYSDISC	0.47	AntiCP	AntiCP	0.47	-	0.47
h	VPSEEIVLADQGSAGGACGVT RHPGLPVVPYSDISC	0.45	AntiCP	AntiCP	0.45	-	0.45

### Docking and virtual screening to find interacting the amino acids

**Fig. 6:**
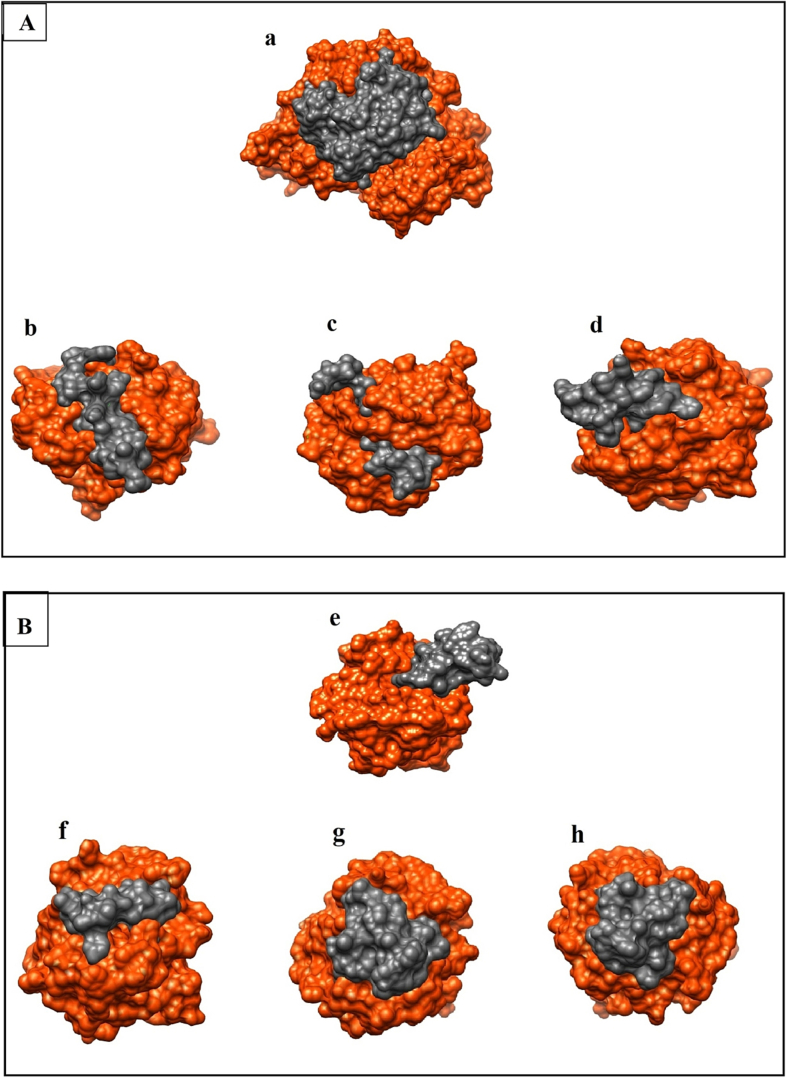
(A) Pockets/cavities of GP63 (a: interaction of GP63/MMP2, b, c, and d: interactions of anticancer peptides of GP63/MMP2). (B) Common peptides of GP63 and MMP2 (e: continuous peptide with no anticancer property, f: predict peptide of GP63/MMP2 with anticancer property, and g: design peptides of GP63/MMP2), using Chimera software. The pockets/cavities are delineated as orange-red is GP63; the MMP2 molecule is depicted as dim gray.

GP63/MMP2 interaction, the binding affinity was in Table 6. Obtained findings from the ClusPro web server indicate that GP63 and its anticancer peptides interact very closely in the central pockets of MMP2 (Fig. 6A). Then docking of the common peptides as a continuous peptide predicted and designed peptides with anticancer property shown in (Fig. 6B). We selected the model with the lowest energy (the first and best model based on the ClusPro web server). Table 6 shows the values of all HDOCK scores expressed as ITScore-PP.

**Table 6:** Center and lowest energy for experimentally verified ACPs of cancer cells in GP63 and common peptides of GP63/MMP2, b, c, and d: anticancer peptides of GP63/MMP2, e: continuous peptide, f: predict peptide, g, and h: design peptides of GP63/MMP2 after docking with MMP2 by ClusPro and docking scores and ligand RMSD (Ǻ) by HDOCK server.

ID sequences		ClusPro server		Hdock server	
		Representative	Weighted Score	Docking Score^a^	Ligand RMSD (Å)
b	LIPQAVQLHTERLKVQQVQGKWKV	Center	–898.5	–205.00	103.31
		Lowest Energy	–928.2		
c	EEGVLAWATTCQTFSDGHPAV	Center	–930.0	–211.96	98.01
		Lowest Energy	–1150.9		
d	HPGLPPYWQYFTDPSLAGVSAFMDYCPVVV	Center	–926.9	–253.65	80.56
		Lowest Energy	–1021.2		
e	VPSEEGVLADQGSAGGACGVTRHPGLPVVPYSDISC	Center	–545.6	–181.52	18.57
		Lowest Energy	–678.3		
f	GACGVTRHPGLPVVPYSDISC	Center	–698.7	–206.47	87.94
		Lowest Energy	–920.5		
g	VPSEERVLADQGSAGGACGVTRHPGLPVVPYSDISC	Center	–576.8	–219.62	73.18
		Lowest Energy	–709.8		
h	VPSEEIVLADQGSAGGACGVTRHPGLPVVPYSDISC	Center	–625.0	–193.87	91.99
		Lowest Energy	–684.7		

a ITScore-PP

**Table 7:** Prediction 3D structures for experimentally verified ACPs of cancer cells in GP63 and common peptides of GP63/MMP2, b, c, and d: anticancer peptides of GP63/MMP2, e: continuous peptide, f: predict peptide, g and h: design peptides of GP63/MMP2 by I-TASSER MODEL.

ID	Sequences	C-score	Estimated TM-score	Estimated RMSD
b	LIPQAVQLHTERLKVQQVQGKWKV	–1.12	0.57±0.14	3.4±2.3Å
c	EEGVLAWATTCQTFSDGHPAV	–0.81	0.61±0.14	2.5±1.9Å
d	HPGLPPYWQYFTDPSLAGVSAFMDYCPVVV	0.10	0.73±0.11	1.6±1.4Å
e	VPSEEGVLADQGSAGGACGVTRHPGLPVVPYSDISC	–2.11	0.46±0.15	6.2±3.8Å
f	GACGVTRHPGLPVVPYSDISC	–1.69	0.51±0.15	4.2±2.8Å
g	VPSEERVLADQGSAGGACGVTRHPGLPVVPYSDISC	–2.25	0.45±0.14	6.5±3.9Å
h	VPSEEIVLADQGSAGGACGVTRHPGLPVVPYSDISC	–2.21	0.45±0.15	6.4±3.9Å

TM-score has the value in [0, 1], where 1 indicates a perfect match between two structures. Following strict statistics of structures in the PDB, scores below 0.17 correspond to randomly chosen unrelated proteins, whereas structures higher than 0.5 generally assume the same fold in SCOP/CATH (kcal/J).

**Table 8:** 2D interaction plot analysis of hydrophobic interactions and hydrogen bonds of GP63 and MMP2, b, c, and d: anticancer peptides of GP63/MMP2, e: continuous peptide, f: predict peptide, g and h: design peptides of GP63/MMP2.

	b	c	d	E	f	g	h
Hydrophobic interactions	PHE4 PHE86 TYR73 HIS130 ALA139 ILE141	LEU81 LEU116 TYR73 LEU82 LEU116 PHE4 PHE148 ALA83 ALA139 ALA121	LEU81 PHE4 PHE86 HIS84 VAL92	LEU81 TYR73 HIS84	LEU81 PHE4 TYR73 ASP76 PRO140	LEU81 TYR73 ASP79 GLY80	LEU81 PHE4 PRO5 PRO140 TYR73 PHE75 HIS84 HIS124
Hydrogen bonds	LEU81 GLU29 ARG6 TYR73 ASP76 GLY80 HIS130 TYR142 THR143	LEU116 HIS120 ALA139 PRO140 TYR143 TYR145	LEU82 ASP76 ALA83 ALA85 HIS84 PRO140	ARG6 GLY80 ALA85 HIS84 HIS124 HIS130	GLU129 ARG6 TYR73 ASP76 GLY80 ALA87 VAL92 PRO140	ASP76 ASP79 GLY80 TYR112 HIS130	GLU129 GLY72 ALA83 HIS120 HIS124 PRO140

### 2D interaction plots of protein–ligand complexes

**Fig. 7:**
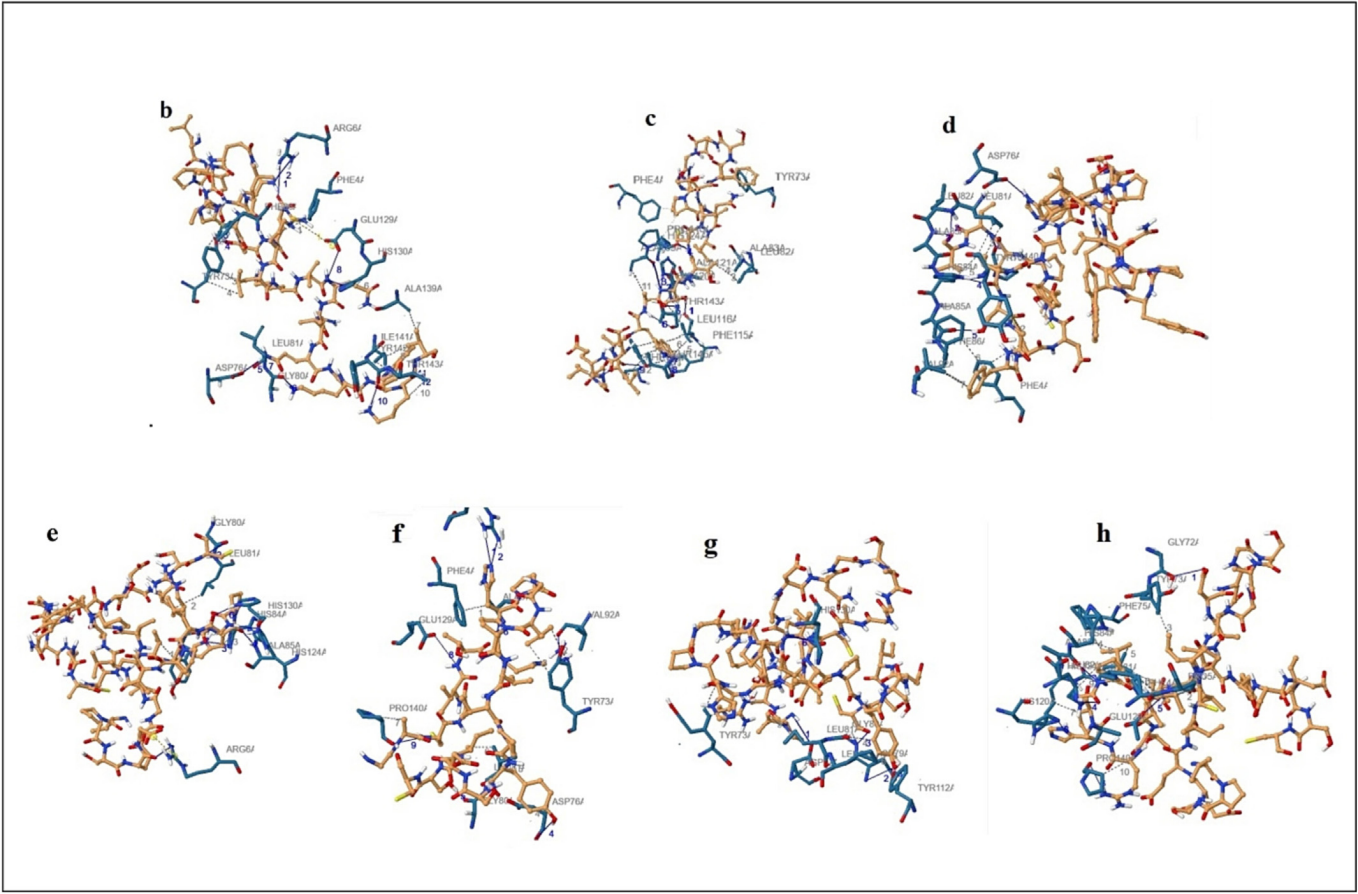
Ligands of GP63 (b, c, and d: ligands of anticancer peptides of GP63 and MMP2) and ligands of peptides of GP63 and MMP2 (e: continuous peptide with no anticancer property, f: predict peptides of GP63 and MMP2 with anticancer property, g and h: design peptides of GP63 and MMP2 with the anticancer property) by PLIP server.

In the present study, the amino acids involved in the interplay between the central pocket of GP63 and MMP2 were determined. After docking, the results of 2D interaction analysis exhibited that in terms of hydrogen interactions and hydrogen bonds mostly between LEU 81 and LEU 116 as shown as functional residues in Hotspot Wizard 3 server results. Fig. 7 shows ligands of GP63 anticancer peptides common peptides as a continuous peptide, predicted, and designed peptides, hydrophobic interactions and hydrogen bonds of peptides summarized in Table 8.

## Discussion

Protein secondary structure predicted by the SOPMA server indicates that alpha helixes consist of the most significant portion (30.07%), random coils place second (51.33%), and extended strands (13.95%) place third, and beta-turn (4.65%) place fourth. Because 3D structures were not available in standard databases, models built by different servers and software were evaluated in terms of structural quality. Although the algorithms for predicting the 3D structure of proteins are considered in bioinformatics modeling^28^; however, it has been observed that the predicted models could have significant differences and errors from their native structures.^29^ In this study, the 3D structure of GP63 was determined by various *in silico* modeling methods. For the first time, the selected peptides were predicted and designed with AntiCP 2.0 server and firmed by I-TASSER modeling. The ModWeb server with the highest Z score (–1.83) was selected for further analysis in this study.

The QMEAN Z-score is an independent protein size measure of the absolute quality of a protein structure model by comparing it to X-ray crystallography reference structures.^30^ Scores assessed by Qmean and Prosa servers confirmed this selection. Accordingly, the necessity of the refining process is inevitable.^31^ The purpose of this process is to produce 3D structures that retain closer pivotal properties (topology, hydrogen bonds, and the side-chain position) to the native state of the protein, which dramatically increases the quality of the predicted models ultimately.^32^^,^^33^ All loop regions were refined and re-evaluated by the ModLoop software none of the modified loop files showed residue changes in the GP63 protein loop regions compared to the initial PDB files. The refinement process applied for GP63 using 3Drefine. We attempted to predict the most accurate and reliable structures for GP63. The results of Ramachandran plot analysis for GP63 (MODWEB MODE) illustrated that the refined structures are very reliable compared to the initially predicted structures (99.036% and none of the residues are in the favored, allowed, and outlier regions, respectively).

The assessments of the functional amino acids evidence that Leu81, Leu116, and Glu129 residues were conserved amino acids. Solvent accessible surface area or volume (S.A.) and mouth M.S. area were 105.795 and 57.181. Interaction of GP63 and MMP2 with the selected structures, specifically with the target amino acids, was evaluated. A key *in silico* technique is molecular docking, which predicts the mechanism of interaction between a small ligand and a target protein for a known binding site. The strength and affinity with which a molecule attaches to the pocket of a target protein are measured by binding energy.^34^ The validity of the GP63 interactions was determined using molecular docking research. The center and lowest energy for experimentally verified anticancer peptides (ACPs) of cancer cells in GP63, and expected standard peptides of GP63/MMP2 were between –545 and –1025. Jairo Mercado-Camargo *et al*. used Auto Dock Vina for Docking of Flavonoids of GP63 and showed that compounds like flavonoids, chalcones, and bioflavonoids showed great affinity to the leishmanolysin protein from *L. major.*^14^ After docking, the 2D interaction diagrams were evaluated. Prediction for experimentally ACPs of cancer cells in GP63 peptides-based therapy has been widely used nowadays, especially in treating various cancers.^35^

In this study, we used 3 peptides of GP63 based on the AntiCP 2.0 server with scores of 0.63, 0.53, and 0.49, and common peptides of GP63/MMP2 (continues peptide: mean the thoroughly selected peptide after docking with non-anticancer effect), predicted with 0.58 score and designed peptides with 0.47 and 0.45 scores by AntiCP 2.0 server. In addition, analysis of the physicochemical properties of the anticancer peptide considered by AntiCP showed that the mean hydrophobicity score was high. Conversely, the mean hydrophilicity score was lower, and these peptides were used in further experimental studies. In addition, the 2D interaction analysis exhibited hydrogen interactions and hydrogen bonds mostly between LEU 81 and LEU 116, as shown as functional residues in HotSpot Wizard 3 server results.

Various studies have shown that some structures, typically in an alpha-helical conformation, can rapidly destroy cancer cell membranes by toroidal-like pore formation. Due to the folding of the predicted structures (Fig. 3, by I-TASSER webserver) with various approaches, our study showed that the resulting structure is one of the proposed structures.^36^ Meanwhile, one of the most critical aspects of these peptides is stapled engineered peptides shown in various studies to cause cell death.^37^

However, peptides with anticancer properties derived from peptides of microorganisms, with cationic or amphipathic properties are host defense peptides that play an essential role in protecting innate immunity against viruses, bacteria, and fungi. They disrupt the action of microbial cell membranes by interacting with negatively charged phospholipids. In addition, these peptides can bind to negatively charged phosphatidylserine moieties that are selectively located on the outer surface of cancer cell plasma membranes, damaging the cancer cell.^38^

It is for the first time that used anticancer peptides of GP63 and the predicted and designed peptides after docking GP63/MMP2. The antileishmanial and anticancer peptide research topics, we believe, best demonstrate the multidisciplinary nature of peptide research. The advancement of cancer and/or *Leishmania* therapies necessitates the integrated research method outlined in this body of work.

## Conclusion

The present study shows that prior *in silico* designing of anticancer peptides is advantageous for their synthesis and characterization. Our investigation was completed using comprehensive web servers or online resources to create peptide libraries with the integrated machine-learning classifier, which led to the discovery of novel ACPs. This method takes less time and is more cost-effective.

## Ethics approval and consent to participate

This investigation was supported by the Research Center of Tropical and Infectious Diseases, 10.13039/501100004621Kerman University of Medical Sciences, Kerman, Iran (with Reg. No. 400001187). It was approved by the ethical committee of Kerman University of Medical Sciences. The Ethics approval code is IR.KMU.REC.1400.667.

## Funding

Not applicable.

## Declaration of interests

The authors declare the following financial interests/personal relationships which may be considered as potential competing interests:

Ali Afgar reports was provided by Kerman University of Medical Sciences. Ali Afgar reports a relationship with Kerman University of Medical Sciences that includes.
